# Hydrophobia

**Published:** 1857-10

**Authors:** 


*Hydrophobia.*

The following extract in relation to this disease has been cut from some recent paper, and sent to us by a medical friend; and we cheerfully give it a place in the *Journal*, although, if we are not mistaken, the same facts were published several years ago in most of the medical journals then existing, both in Europe and America.

*A cure for Hydrophobia.*—We have received from a gentleman at Berlin the following important statement of the mode of cure practiced in Ukraine for the bite of a mad dog. It is translated from the *Berlin State Gazette* (No. 20) of the 14th of February, 1822, and does certainly seem entitled to the fullest consideration of all medical men. That the knowledge of this remedy may be extensively known, and further put to test by experience, we hope it will be copied into every journal throughout the country.

When Mr. Marochetti, an operator in the hospital at Moscow, was in the Ukarine in 1813, in one day *fifteen* persons applied to him for cure, having been bitten by a mad dog. Whilst he was preparing the remedies, a deputation of several old men made its appearance, to request him to allow a peasant to treat them, a man who for some years enjoyed a good reputation for his cures of hydrophobia, and of whose success Mr. Marochetti had already heard so much. He consented to their request under these conditions:—1. That he, Mr. Marochetti, should be present at everything done by the peasant. 2. In order that he might be fully convinced that the dog was really mad, he, Mr. M., should select one of the patients, who should only be treated according to the medical course usually held in estimation. A girl of six years old was chosen for this purpose. The peasant gave his fourteen patients a strong decoction of the 'Summit' and Fl. Genista Lutea tincturæ (about a pound and a half daily,) and examined twice a day under the tongue, where, he stated, *small knots*, containing the poison of the madness, must form themselves. As soon as these small knots actually appeared, and which Mr. Marochetti himself saw, they were opened and cauterized with a red-hot needle; after which the patient gargled with the decoction of the Genista. The result of this treatment was that all the fourteen (of whom only two, the last bitten, did not show these knots,) were dismissed perfectly cured at the end of six weeks, during which time they drank this decoction. But the little girl who had been treated according to the usual methods, was seized with hydrophobic symptoms on the seventh day, and was dead in eight hours after they first took place. The persons dismissed as cured were seen three years afterward by Mr. Marochetti, and they were all sound and well. Five years after this circumstance (in 1818) Mr. Marochetti had a new opportunity, in Podolia, of *confirming* this important discovery. The treatment of twenty-six persons who had been bitten by a mad dog was confided to him—nine were men, eleven women, and six children. He gave them at once a decoction of the Genista: and a diligent examination of their tongues gave the following result:—Five men, all the women, and three children had the small knots already mentioned—those bitten most, on the third day; others on the fifth, seventh and ninth; and one woman who had been bitten only very superficially, on the twenty-first day. The other seven, who showed *no* small knots, drank the decotion Genista six weeks, and all the patients were cured.

In consequence of these observations, Mr. Marochetti believes that the hydrophobic poison, after remaining a short time in the wound, fixed itself for a certain time under the tongue, at the opening of the ducts of the submaxillary glands, which are at each side of the tongue-string, and there forms those smaller knots, in which one may feel with a probe a fluctuating fluid, which is the hydrophobic poison. The usual time for their appearance seems to be between the third and ninth day after its bite; and if they are *not* opened after the first twenty-four hours of their formation, the poison is re-absorbed by the body, or into the body, and the *patient is lost beyond the power of cure.* For this reason Mr. Marochetti recommends that such patients should be continued for six weeks, during which time they should take daily 1½ lb. of the decoct. genist. (or four times a day the powder, 1 drach pro. dost.) If the knots do not appear in this time, no madness is to be apprehended; but, as soon as they show themselves, they s ould be opened with a lancet, and then cauterized, and the patient should gargle assiduously with the above-mentioned decoction.

"We hasten," says the *Berlin Gazette*, "to communicate to our readers this important discovery (which we borrow from the 'Petersburg Miscellaneous Treatises in the Realm of Medical

Science for 1821,') which certainly deserves the full attention of all medical practitioners, and which, if confirmed by experience, may have the most beneficial results."

